# Comparison of Serum Bisphenol A Concentrations in Mice Exposed to Bisphenol A through the Diet versus Oral Bolus Exposure

**DOI:** 10.1289/ehp.1003385

**Published:** 2011-06-06

**Authors:** Paizlee T. Sieli, Eldin Jašarevic´, Denise A. Warzak, Jiude Mao, Mark R. Ellersieck, Chunyang Liao, Kurunthachalam Kannan, Séverine H. Collet, Pierre-Louis Toutain, Frederick S. vom Saal, Cheryl S. Rosenfeld

**Affiliations:** 1 Department of Biomedical Sciences,; 2 Bond Life Sciences Center,; 3 Department of Biological Sciences,; 4 Interdisciplinary Neuroscience Program,; 5 Division of Animal Sciences, and; 6 Agricultural Experiment Station, University of Missouri, Columbia, Missouri, USA; 7 Wadsworth Center, New York State Department of Health, and Department of Environmental Health Sciences, School of Public Health, State University of New York at Albany, Albany, New York, USA; 8 INRA, UMR1331, Toxalim (Research Centre in Food Toxicology), Toulouse, France; 9 Université de Toulouse, INP, ENVT, UPS, EIP, Toulouse, France

**Keywords:** bioavailability, BPA, chronic exposure, deconvolution analysis, endocrine disruptor, food effect, oral bolus, pharmacokinetic analysis

## Abstract

Background: Bisphenol A (BPA) is a widely produced endocrine-disrupting chemical. Diet is a primary route of exposure, but internal exposure (serum concentrations) in animals and humans has been measured only after single oral bolus administration.

Objective: We compared serum concentrations of BPA over a 24-hr period after oral bolus administration or *ad libitum* feeding in mice and assessed for buildup with dietary exposure.

Methods: Adult female mice were administered [dimethyl-*d*_6_]-BPA (BPA-*d*_6_) as a single oral bolus (20 mg/kg body weight) or fed a diet containing 100 mg BPA-*d*_6_/kg feed weight *ad libitum* for 1 week. Serum concentrations were analyzed using isotope dilution liquid chromatography coupled with electrospray tandem mass spectrometry and compared between exposure groups over the first 23 hr and after 7 days of dietary exposure.

Results: Maximum concentration (*C*_max_) for BPA-*d*_6_ during the first 24 hr was reached at 1 hr and 6 hr for oral bolus and diet groups, respectively. Relative BPA-*d*_6_ bioavailability (unconjugated BPA-*d*_6_) was higher in diet-exposed mice than in the bolus group despite a relative lower absorption, a phenomenon consistent with an inhibitory effect of food on first-pass hepatic metabolism. In mice with ongoing dietary exposure, unconjugated BPA-*d*_6_ was higher on day 7 than on day 1.

Conclusions: This is the first report of serum BPA concentrations in an animal model exposed to this chemical via the diet. Although bolus administration of BPA-*d*_6_ led to peak concentrations within 1 hr, *C*_max_ for diet-exposed mice was delayed for several hours. However, bolus administration underestimates bioavailable serum BPA concentrations in animals—and presumably humans—than would result from dietary exposure. Exposure via diet is a more natural continuous exposure route than oral bolus exposure and is thus a better predictor of BPA concentrations in chronically exposed animals and humans.

Bisphenol A (BPA) was first developed in the 1930s and is presently used in the manufacture of many polycarbonate plastic containers (including baby bottles and reusable water bottles), dental sealants, metallic food cans, paper, and cardboard items (Biedermann et al. 2010; Galloway et al. 2010; He et al. 2009). Because this chemical was assumed to be relatively harmless, demand for these products has increased, and so has the manufacture of BPA, which is now produced in amounts exceeding 8 billion pounds/year, with little signs of diminution of output (Bailin et al. 2008). BPA is stable in sediment and detectable in almost all bodies of water (Environment Canada 2008). Thus, exposure of wildlife and humans to BPA is inevitable, likely to continue, and even to increase (Vandenberg et al. 2009). Human and animal contact with BPA can occur through various sources, and whether diet is the primary source of exposure remains unresolved (Stahlhut et al. 2009). Regardless, > 90% of people in the United States have measurable levels of BPA (Calafat et al. 2008), and there is no reason to suppose that the reach of BPA is not global (Vandenberg et al. 2010a).

The overarching question concerns the total amount of BPA that most humans are subjected to on a daily basis. An attendant question is whether these exposures lead to adverse outcomes. Although a handful of studies have tried to address this question by measuring serum and urinary concentrations of BPA in human populations, great variability exists in the exact estimates [Food and Drug Administration (FDA) 2008; Vandenberg et al. 2007; vom Saal et al. 2007]. For instance, the FDA estimated that the daily BPA exposure for adults is 0.16 μg/kg/day (FDA 2008). However, based on the available data at the time, a conference sponsored by the National Institute of Environmental Health Sciences in 2007 predicted that internal exposure (plasma or serum concentrations) in humans is > 35 mg/day (~ 500 μg/kg/day)  (Vandenberg et al. 2007; vom Saal et al. 2007). This prediction has been updated (Vandenberg et al. 2010a) to reflect mounting evidence suggesting that human exposure to BPA can occur through routes other than diet and water consumption (Gies et al. 2009; Vandenberg et al. 2010b).

A major hurdle in estimating human exposure to BPA is accounting for all of the potential routes of exposure, even though contaminated food and beverages are still considered the dominant source for this chemical (Galloway et al. 2010; Vandenberg et al. 2009; Willhite et al. 2008). For example, BPA exposure might also occur through less-explored routes, including dermal contact with thermal (carbonless) receipts, inhalation of household dusts, and cigarette smoke (Biedermann et al. 2010; Galloway et al. 2010; He et al. 2009). Only one published study to date has examined the elimination of BPA from blood after a single oral administration to volunteer human subjects, and this study failed to detect active BPA in the serum of these individuals (Volkel et al. 2002), most probably because concentrations fell below the detection limit of the relatively insensitive assay employed (Taylor et al. 2011; Vandenberg et al. 2009). Rather than recognize the potential limitation of that study, some investigators have used the data to conclude that unconjugated BPA is so rapidly metabolized and/or cleared that it is relatively harmless to humans (Dekant and Volkel 2008; Willhite et al. 2008).

Rodent models have been criticized as inappropriate to calculate human BPA exposures based on the prediction of species differences in metabolism of BPA (Dekant and Volkel 2008), although this conclusion was disputed by an expert panel of the Food and Agriculture Organization of the United Nations and the World Health Organization (2010). Although both adult rodents and primates use glucuronidation of BPA through uridine 5´-diphospho-glucuronosyltransferases as part of their phase II metabolism system, the primary mechanism through which BPA is cleared from blood differs, with urinary excretion being the primary route in primates versus the biliary–fecal route in rodents (Inoue et al. 2005; Sakamoto et al. 2002). BPA might also be metabolized by sulfonation, but this form accounts for only a very minor component across various adult animal species (Balakrishnan et al. 2010; Kurebayashi et al. 2003; Pottenger et al. 2000). Enterohepatic recirculation of BPA is less prominent in primates than in rodents, but even in rats and mice, enterohepatic recirculation is not a major factor (Doerge et al. 2010a; Taylor et al. 2011). A recent study that performed a side-by-side analysis of the serum concentrations of BPA in CD1 mice and rhesus monkeys (*Macaca mulatta*) that had, in each case, received an oral bolus of the chemical concluded that the clearance of unconjugated BPA over 24 hr was comparable in the two species (Taylor et al. 2011) and similar to what had been observed in a second macaque species, *Macaca fascicularis* (Tominaga et al. 2006). Thus, by these criteria, mice appear to be an acceptable animal model to predict the pharmacokinetic of BPA in nonhuman primates and potentially, by extrapolation, in humans (Gies et al. 2009; Taylor et al. 2011).

Both in the single human study by Volkel et al. (2002) and in the various trials performed on nonhuman primates and rodents, serum concentrations of conjugated and unconjugated BPA have invariably been measured after administering a single dose of the chemical as either an oral bolus or via subcutaneous injection (Doerge et al. 2010a, 2010b; Taylor et al. 2011; Vandenberg et al. 2009), whereas continuous exposure through the diet seems more likely to mimic exposures outside the laboratory. Moreover, dietary BPA exposure would be a more appropriate and convenient route than bolus exposure for studies of the developmental effects of *in utero* BPA exposure on offspring and adults. Although pregnant mice fed a diet containing BPA have given birth to offspring with epigenetic (Dolinoy et al. 2007) and behavioral abnormalities (Cox et al. 2010), neither study measured serum concentrations of BPA during pregnancy. Finally, it remains controversial whether the quantities of BPA supplied to mice in these studies reflect exposure levels that might be expected to occur outside of the laboratory. In the present study, we measured serum concentrations of BPA in mice exposed through natural feeding behavior to more precisely characterize circulating concentrations resulting from dietary exposure and compared concentrations with those in mice exposed through single oral bolus exposure, as in previous studies (Doerge et al. 2010a, 2010b; Taylor et al. 2011). Instead of using BPA, we employed the isotopically tagged form, [dimethyl-d_6_]-BPA (BPA-*d*_6_; C/D/N Isotopes Inc., Quebec, Canada), to ensure that only the BPA provided experimentally was assayed.

## Materials and Methods

*Animals.* All animal experiments were approved by the University of Missouri Animal Care and Use Committee and performed in accordance with National Institutes of Health animal care and use guidelines (Institute of Laboratory Animal Resources 1996). All animals were treated humanely and with regard for alleviation of suffering. Adult (10–12 weeks of age) C57Bl/6J female mice were purchased from Jackson Labs (Bar Harbor, ME, USA). On arrival, animals were placed on AIN93G diet (Harlan Teklad, Madison, WI, USA) [see Supplemental Material, Table 1 (http://dx.doi.org/10.1289/ehp.1003385)], and their food consumption and body weights (BWs) (mean ± SE, 19.1 ± 0.5 g) were measured daily. Mice were maintained on a 12-hr dark:12-hr light cycle with lights out at 1900 hours. To minimize background BPA exposure, mice were housed in polypropylene cages and provided glass water bottles. Their water was stringently purified by a reverse osmosis and carbon filtration system and did not contain detectable BPA.

**Table 1:** Pharmacokinetics parameters of BPA‑*d*_6_ obtained after NCA of unconjugated and total BPA‑*d*_6_ serum concentrations in mice over a 24-hr period after exposure via oral bolus or diet.

Oral bolus (20 mg/kg BW)			Diet (13 mg/kg BW)*a*		
Pharmacokinetic parameter	Unconjugated	Total	Unconjugated	Total
*C*_max_ (ng/mL)		21.0 ± 3.9		1636.5 ± 642.6		18.8 ± 4.4		802.2 ± 126.6
AUC_0–24hr_ (ng.hr/mL)		201.0 ± 20.6		21979.3 ± 3813.5		147.8 ± 26.7		11547.3 ± 1219.5
Average 24-hr concentration (ng/mL)		8.3		915.8		6.1		481.1
AUC_0–24hr_ total/unconjugated BPA‑*d*_6_		109				79		
**a**Estimated ingested dose based on food consumption.								

*BPA-*d*_6_ treatments.* Based on the widely accepted studies demonstrating that BPA disposition is linear over a wide range of doses, including in humans (Doerge et al. 2010a; Taylor et al. 2011; Teeguarden et al. 2011; Vandenberg et al. 2007), our experiments were confined to a single test dose that would provide detectable and accurate measurements for mice exposed to BPA-*d*_6_ through diet or oral bolus exposure. At 1900 hours (the beginning of the dark cycle), one group of nonfasted mice that had not yet initiated their nocturnal feeding received a single oral bolus of 20 mg/kg BW BPA-*d*_6_ in an exceedingly small volume (40 μL) of tocopherol-stripped corn oil (MP Biomedicals, LLC, Solon, OH, USA). These mice continued to receive AIN93G diet after exposure. This oral bolus dose was chosen to approximate the amount of BPA-*d*_6_ that mice on a diet containing 100 mg BPA-*d*_6_/kg feed weight [see Supplemental Material, Table 2 (http://dx.doi.org/10.1289/ehp.1003385)] had been predicted to consume in a 24-hr period based on preliminary data and data from another published study using the same strain of mice (Cox et al. 2010).

For the BPA-*d*_6_ diet–exposed group, the food placed at the beginning and remaining at the end of each time point was weighed to calculate the amount consumed. Each mouse was weighed immediately before it was killed for blood collection. Two to four mice for each time point and group were caged together to reduce space. To account for the varying numbers of mice in the cage, each cage was considered a unit, and the total consumption for each cage was divided by the average weight of the mice in this cage.

Serum samples from mice that had not been administered BPA-*d*_6_ were collected from females (*n* = 12) maintained on AIN93G diet beginning at 1900 hours, which is when mice normally begin nocturnal feeding at the beginning of the dark phase of the dark:light cycle (Kavaliers et al. 1985). Representative mice from the diet and oral bolus groups (*n* = 8 for each time point) were culled and cardiac blood was collected 1 hr, 4 hr, 6 hr, 11 hr, 24 hr, and 7 days (168 hr) after initiating exposure to BPA-*d*_6_ at 1900 hours. For additional information on BPA-*d*_6_ treatments, see Supplemental Material, p. 3 (http://dx.doi.org/10.1289/ehp.1003385).

*Analysis of unconjugated and conjugated BPA-*d*_6_ in mouse serum samples.* BPA-*d*_6_ serum concentrations were measured as described previously (Padmanabhan et al. 2008) but with some modifications. The mouse serum samples were divided into two aliquots (each of 150–200 μL) for the analysis of free and total BPA-*d*_6_, respectively. A reference standard [5 ng deuterated 16-BPA (BPA-*d*_16_)] was included as a quality control to validate the method, as the inclusion of BPA-*d*_16_ into the test serum samples served as an internal control to estimate recovery through the analytical steps. Analyte separation and detection were carried out by using an Agilent 1100 series HPLC interfaced with an Applied Biosystems API 2000 electrospray MS/mS mass spectrometer (Applied Biosystems, Foster City, CA, USA). Additional information is provided in Supplemental Material, pp. 3–5 (http://dx.doi.org/10.1289/ehp.1003385).

*Statistical analysis.* We used analysis of variance and SAS software (version 9.2; SAS Institute Inc., Cary, NC, USA) to analyze differences in serum concentrations of unconjugated and total BPA-*d*_6_ within diet and oral bolus groups. However, because there were heterogeneous variances, tests for significance were performed after log_10_ transformation. The linear statistical model was a two by seven factorial (two treatments, seven time points). If main effects were statistically significant at *p* < 0.05, mean differences were determined using Fisher’s least significant difference. All data are presented as mean ± SE.

*Pharmacokinetic analysis.* Unconjugated and total serum concentration–time profiles after oral and diet BPA-*d*_6_ exposure were analyzed with a noncompartmental analysis (NCA) using WinNonlin (WinNonlin® Professional, version 5.3; Pharsight Corporation, Cary, NC, USA). We calculated area under the curve (AUC) up to the last quantifiable serum concentration [i.e., AUC_(0–24hr)_], using the linear trapezoidal rule. For additional information on NCA analysis, see Supplemental Material, p. 5 (http://dx.doi.org/10.1289/ehp.1003385). The sparse data option of WinNonlin was used, allowing computation of the different SEs associated with estimated parameters (see Supplemental Material, Tables 3–5).

We analyzed unconjugated BPA-*d*_6_ serum concentrations after oral exposure with a compartmental analysis using a monocompartmental model without lag time. Pooled data were fitted using the following equation (the so-called Bateman equation):


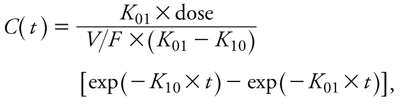
[1]

where *C*(*t*) is the pooled BPA-*d*_6_ serum concentration at time *t*, *F* is the unknown bioavailability of BPA-*d*_6_, dose is the BPA-*d*_6_ dose, *V* is the volume of distribution (milliliters per kilogram), *K*_01_ (per hour) is the first-order rate constant of absorption and *K*_10_ (per hour) is the first-order rate constant of elimination. *V/F*, *K*_10_, and *K*_01_ were estimated. Iterative reweighting was used during minimization process, that is, the data were weighted by the inverse of the observed value (1/*Y*_obs_) [see Supplemental Material, Figure 1 (http://dx.doi.org/10.1289/ehp.1003385)]. The goodness of fit of the model was assessed by using least-squares criteria and visual inspection of residuals. Weighted residuals are presented in Supplemental Material, Figure 2. For additional information on compartmental analysis, see Supplemental Material, p. 6.

**Figure 1:**
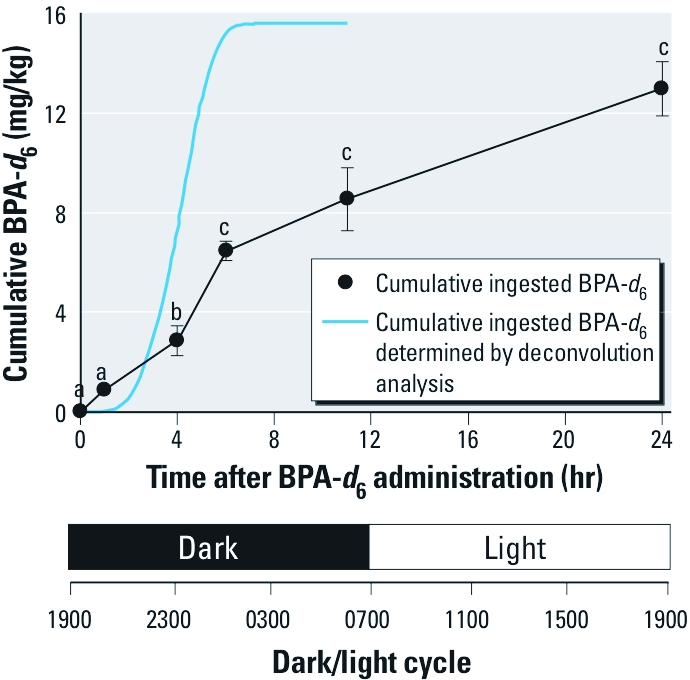
Cumulative diet exposure to BPA‑*d*_6_ versus time and night/day cycle estimated by amount of food consumed and by deconvolution. Deconvolution analysis was performed by using the oral bolus administration as a reference to evaluate the *in vivo* BPA‑*d*_6_ input rate (BW) over 11 hr (i.e, during the night). Time points with different superscripts are significantly different from each other based on log_10_ scale analysis (*p* < 0.01).

**Figure 2:**
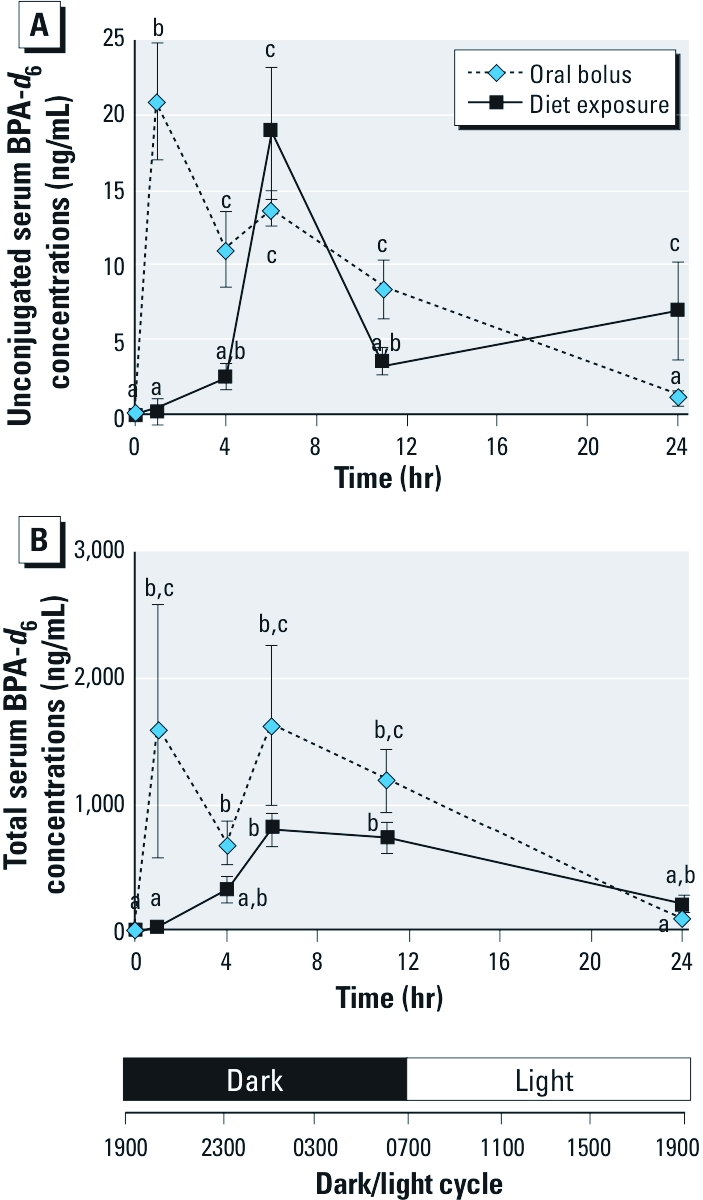
Arithmetic plot of the mean (± SE) serum concentrations of unconjugated BPA‑*d*_6_ (*A*) (and the total BPA‑*d*_6_ serum concentrations (*B*) versus time and versus night/day cycle after a single oral bolus of BPA‑*d*_6_ at 20 mg/kg BW or after exposure to BPA‑*d*_6_ at 100 mg/kg feed weight (13 mg/kg BW in the first 24 hr; see Figure 1) in mice. For the oral bolus, *C*_max_ for unconjugated BPA‑*d*_6_ and total BPA‑*d*_6_ occurred 1 hr after the treatment (2000 hours). In contrast, *C*_max_ was not obtained until 6 hr after treatment (0100 hours) in the diet-exposed group. Within each treatment group, values with different superscripts are significantly different (*p* < 0.05).

The relative bioavailability between the two conditions of administration was calculated from the following equation:


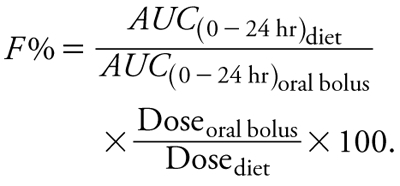
[2]

*AUC*_(0–24hr)oral bolus_ and *AUC*_(0–24hr)diet_  (the AUC of unconjugated serum BPA) was estimated by noncompartmental analysis, and Dose_oral bolus_ and Dose_diet_ were the actual nominal dose and dose ingested through the diet, respectively, with the diet dose estimated by food consumption. We also used Equation 2 to estimate the extent of BPA-*d*_6_ absorption replacing unconjugated BPA-*d*_6_ AUCs by the corresponding total BPA-*d*_6_ AUCs.

We used deconvolution to evaluate *in vivo* drug release and delivery when data from a known drug input were available (i.e., the kinetics after the oral bolus BPA-*d*_6_ administration). The BPA-*d*_6_ input rate evaluated when BPA-*d*_6_ was administered in the diet corresponded to the *in vivo* BPA-*d*_6_ release from food followed by a BPA-*d*_6_ delivery to the general systemic circulation. In this experiment, deconvolution was used to evaluate the *in vivo* BPA-*d*_6_ input rate (milligrams per hour) into blood of mice fed during the night. Data from 0 to 11 hours (i.e., only data collected during the nocturnal period) were considered. The reference BPA input was obtained from the bolus oral administration for which the input into the digestive tract was instantaneous (drenching). The deconvolution analysis was performed using the mean of pooled unconjugated BPA-*d*_6_ serum concentrations after diet exposure over time. More details and results of these analyses are included in Supplemental Material, pp. 5–8, 11–18, and 21; Tables 3–7; and Figures 3 and 4 (http://dx.doi.org/10.1289/ehp.1003385).

## Results

*Measurement of external cumulative exposure rate to BPA-*d*_6_ in diet-exposed mice.* By knowing the weight of BPA-*d*_6_–supplemented diet provided at the outset of the experiment, the amounts remaining at each time point of serum collection, and the weight of the mice in each cage at the time of serum collection, we could calculate the cumulative exposure of BPA-*d*_6_ (milligrams per kilogram) in the diet-exposed group (Figure 1). Mice were placed on the BPA-*d*_6_ supplemented diet at 1900 hours (i.e., at the end of their normal light cycle), and within the first 6 hr (i.e., by 0100 hours) the animals had eaten about 50% of the food consumed over 24 hr (Figure 1). By this time point, mice had consumed 6.5 ± 0.4 mg/kg BPA-*d*_6_ (mean ± SE). After 11 hr, consumption had increased to 8.6 ± 1.3 mg/kg and at 24 hr had reached 13.0 ± 1.1 mg/kg. After 7 days of consuming the BPA-*d*_6_–supplemented diet, the total ingested dose was 78.1 ± 0.7 mg/kg rather than the 140 mg/kg expected dose.

No differences in BW were observed among the mice on the two treatment regimens, and overall body mass did not change significantly over the 7-day experimental period. In addition, none of the mice showed outward signs of ill health. These data thus suggest that BPA-*d*_6_ in the feed was well tolerated.

*Measurement of internal exposure to unconjugated BPA-*d*_6_ in serum.* For mice receiving the oral bolus (20 mg/kg BW), maximum concentration (*C*_max_) of unconjugated BPA-*d*_6_ (21.0 ± 3.9 ng/mL, mean ± SE) occurred within 1 hr (i.e., by 2000 hours) of administration of BPA-*d*_6_ (Table 1 and Figure 2A) and declined slowly thereafter, reaching barely detectable concentrations after 24 hours (i.e., at 1900 hours the day after bolus administration). In the diet-exposed group, the estimated dose was 13 mg/kg BW over the first 24 hr, and peak BPA-*d*_6_ concentrations (18.8 ± 4.4 ng/mL) were not observed until 6 hr (0100 hours) after the initiation of the BPA-*d*_6_–supplemented diet (Table 1 and Figure 2A); unconjugated serum BPA-*d*_6_ concentrations declined significantly by 11 hr. However, because *C*_max_ occurred after consuming only 6.5 mg/kg BW of BPA-*d*_6_ (Figures 1 and 2), we derived a scaled *C*_max_ value for dietary exposure consistent with the oral bolus dose of 20 mg/kg BW [i.e., (20 mg/kg ÷ 6.5 mg/kg) × 18.8 ng/mL] resulting in an estimated peak concentration of 57.9 ng/mL for diet exposure. Unconjugated BPA-*d*_6_ serum concentrations collected at 1900 hours from mice on BPA-*d*_6_–containing diet for 7 days were higher than unconjugated BPA-*d*_6_ serum concentrations in mice on this diet for 24 hr (13.2 ± 5.2 ng/mL versus 6.9 ± 3.3 ng/mL, *p* < 0.05), revealing that significant buildup of biologically active BPA-*d*_6_ had occurred during the 7-day exposure period. To compare the oral bolus and diet groups at the same external dose, we scaled the dose of the diet-exposed group to 20 mg/kg BW, and the AUC_(0–24hr)_ was modestly greater (but not statistically significant) in the diet group compared with the oral bolus group (227.4 ± 41.1 and 201.0 ± 20.6 ng-hr/mL, respectively), indicating a relative bioavailability of 113% for the diet group.

Rate constants (initial and terminal rates) were estimated by using compartmental analysis to fitted unconjugated BPA-*d_6_* serum concentrations after oral bolus exposure [see Supplemental Material, Figure 1 (http://dx.doi.org/10.1289/ehp.1003385)]. The terminal half-life of BPA-*d*_6_ was estimated to be 6.4 ± 1.1 hr (mean ± SE, see Supplemental Material, Table 6). Deconvolution analysis for time development of the internal exposure in the diet-exposed group supported our original finding on exposure rate obtained with food consumption. When bolus administration of BPA-*d*_6_ was used as the reference, the total estimated bioavailable BPA-*d*_6_ dose between 0 and 11 hr after the beginning of diet exposure based on deconvolution analysis was 15.6 mg/kg, which is close to the 13 mg/kg BW estimated by actual weighing of ingested food (Figure 1; see also Supplemental Material, Figures 3 and 4). From the deconvolution analysis, it appeared that most of BPA-*d_6_* ingestion was during the first part of the night, and 90% of the bioavailable BPA had been computed to be absorbed into the bloodstream by 5.46 hr after the beginning of the diet exposure (i.e., before 0100 hours the next day) (see Supplemental Material, Table 7).

*Measurement of internal exposure to total BPA-*d*_6_ in serum.* Total BPA is the sum of the unconjugated and conjugated BPA-*d*_6_ in the serum of exposed mice. Concentrations of the conjugated form were up to 70–100 times higher than those of unconjugated BPA (Table 1), which could account for the differences in blood clearance between conjugated and unconjugated forms. In the oral bolus group, peak concentrations (i.e., presumed *C*_max_) of conjugated BPA-*d*_6_ occurred by 1 hr (at 2000 hours) after treatment (1596.7 ± 1006.6 ng/mL, mean ± SE) (Figure 2B). In the oral bolus group, there was a second increase, which in contrast to unconjugated BPA-*d*_6_ data was statistically significant between the 4-hr collections (2300 hours) and the measurements at 6 hr (693.2 ± 176.9 ng/mL versus 1636.5 ± 642.6 ng/mL; *p* < 0.0.01) and at 11 hr (1200.5 ± 252.8) (Figure 2B). By 24 hr, however, total BPA-*d*_6_ had declined markedly in both groups to about 5% of their peak concentrations noted at 1 hr. The *C*_max_ for the diet group (802.2 ± 126.6 ng/mL) was achieved later (at around 6 hr; 0100 hr) than in the bolus group (Figure 2B), thus mirroring the data for unconjugated BPA-*d*_6_ (Figure 2A).When total BPA-*d*_6_ concentrations for diet exposure were scaled to the oral bolus dose [i.e., (20 mg/kg ÷ 6.5 mg/kg) × 802.2 ng/mL], the estimated peak concentration for the diet-exposed group was 2468.3 ng/mL. Total BPA-*d*_6_ concentrations declined after 6 hr in the diet-exposed group (Figure 2B). Concentrations of total serum BPA-*d*_6_ at 24 hr and 7 days in the diet-exposed group were not significantly different (193.9 ± 44.4 ng/mL vs. 359.6 ± 64.23 ng/mL; *p* > 0.05).

Assuming that total BPA-*d*_6_ is formed only by a hepatic first-pass effect (Dekant and Volkel 2008), the AUC for total BPA-*d*_6_ reflects the extent of BPA-*d*_6_ absorption, whereas the amount of unconjugated BPA-*d*_6_ that reaches systemic circulation after escaping hepatic first-pass metabolism reflects the extent of BPA-*d*_6_ bioavailability. The AUC_0–24hr_ for diet exposure and bolus administration at the same external dose were 11547.3 ± 1219.5 and 21979.3 ± 3813.5 ng-hr/mL, respectively, indicating a relative absorption of 81% for the diet group. Thus, less total BPA-*d*_6_ was absorbed after diet exposure than after oral bolus administration. Consequently, the higher bioavailability associated with diet exposure (113%) is presumably explained by postabsorption events rather than by increased BPA-*d*_6_ absorption.

## Discussion

Previous studies have demonstrated that exposure to BPA through diet can induce epigenetic and behavioral changes in mice (Cox et al. 2010; Dolinoy et al. 2007), suggesting that exposure to BPA has measurable biochemical and phenotypic effects in animals. However, to our knowledge, the present study is the first to quantify serum BPA concentrations in any species after exposure through the diet. We chose an external exposure of BPA that was lower than the lowest observed adverse effect level of 50 mg/kg BW/day reported in rodents (Cox et al. 2010) but still within the detection limit of the assay that would yield accurate measurements. Because phase II enzymes are not saturable within many orders of magnitude of human exposure, it is commonly accepted that BPA concentrations in the systemic blood are linear over a wide range of doses (Doerge et al. 2010a; Taylor et al. 2011; Teeguarden et al. 2011; Vandenberg et al. 2007, 2010a), thereby negating the need to measure internal concentrations after much lower doses of BPA-*d*_6_. Intake of BPA-*d*_6_ at the levels used in our study, either as a single bolus dose or through *ad libitum* feeding, allowed us to measure both unconjugated and total BPA-*d*_6_ in the serum of the exposed C57Bl/6J female mice using a combination of HPLC and tandem mass spectrometry. The inclusion of a separate, spiked, deuterated form of BPA, (BPA-*d*_16_) in the collected serum served as an internal control to estimate recovery through the analytical steps. Moreover, the BPA-*d*_6_ consumed could be distinguished from any contaminating compound and the internal control by virtue of the transitions of 233 *m/z* > 215 *m/z* for BPA-*d*_6_ and 241 *m/z* > 223 *m/z* for BPA-*d*_16_. Hence, the method was not only sensitive but specific and accurate for the compounds analyzed.

Although the spacing of sampling times did not permit precise pharmacokinetic profiles to be assessed, it was possible to compare the consequences of ingestion of BPA-*d*_6_ as a bolus versus *ad libitum* consumption in the food, which was the primary goal of the study. Most studies on the effects of BPA in rodents or in nonhuman primates and humans have used a single bolus administration (Doerge et al. 2010a, 2010b; Taylor et al. 2011). We have shown that BPA-*d*_6_ is rapidly metabolized to one or more conjugated forms and that both free and conjugated forms are cleared fairly rapidly from serum after exposure through diet as well as after oral bolus administration. The terminal half-life value for disposition of the unconjugated form of BPA-*d*_6_ after bolus administration was approximately 6 hr, and thus by 24 hr this group had low circulating concentrations of the substance.

An important finding from our experiments is that the bioavailability is higher after diet administration than after bolus administration of BPA-*d*_6_, despite evidence of lower relative absorption after diet administration. This finding may be tentatively explained by a “food-effect,” which has been previously described for several highly extracted drugs (Tam 1993; Wilkinson 1997). Specifically, it has been hypothesized that food transiently inhibits the intrinsic ability of the liver to metabolize highly extracted substances (i.e., chemicals that are preferentially metabolized by the liver), particularly during the absorption phase (Tam 1993; Wilkinson 1997). Thus, consumption of BPA-*d*_6_ in food increased its internal bioavailable concentrations, despite lower BPA-*d*_6_ absorption, relative to oral bolus exposure. When the treatments were initiated at the beginning of the dark phase (1900 hours), neither oral bolus nor diet- exposed groups had commenced their nocturnal feeding. After treatments were initiated, both groups were fed the same amount of a diet that was identical except for the addition of BPA-*d*_6_ [see Supplemental Material, Tables 1 and 2 (http://dx.doi.org/10.1289/ehp.1003385)]. It is unlikely that the very small volume of corn oil used for oral bolus administration affected the results, particularly because maximum concentrations (*C*_max_) also occurred 1 hr postadministration in mice provided an oral bolus of BPA-*d*_6_ during the light phase (versus the dark phase) of the cycle (Taylor et al. 2011). Moreover, although the scaled *C*_max_ (57.9 ng/mL) after administration of BPA-*d*_6_ in the diet was almost three times higher than the *C*_max_ after oral bolus exposure (21.0 ng/mL), the scaled AUC for diet versus oral bolus exposure was only increased by about 13%. This difference between *C*_max_ and AUC is consistent with a transient food effect increasing *C*_max_ but not AUC, which was calculated over a 24-hr period (i.e., before and after the *C*_max_ period). These findings of the effect of food on BPA absorption might account for the spurious or dramatic variations in peak plasma concentrations observed in human biomonitoring studies (Vandenberg et al. 2010b).

Our finding that diet exposure resulted in increased serum concentrations of active BPA-*d*_6_ is relevant to animals and humans, where a significant portion of the total exposure to BPA is believed to occur through diet (Matsumoto et al. 2003). Recently, Teeguarden et al. (2011) demonstrated that in humans there is considerable inter- and intrameal variability in BPA urinary excretion, consistent with an estimated range of exposure from 3.29 to 73.29 μg. However, these findings might also be explained by a food effect, the inhibitory effect of food on first-pass hepatic metabolism. In addition, BPA exposure through food consumption is experimentally more convenient; because it is less stressful than other routes of administration, it may provide a more relevant pharmacokinetic profile by reducing stress-related confounds.

Another potential noteworthy finding is possible bioaccumulation of free BPA when BPA-*d*_6_ is provided through the diet, which has not been observed when BPA is administered as a single bolus (Doerge et al. 2010a; Taylor et al. 2011). For example, in the 24-hr experiment, concentrations of free BPA-*d*_6_ were significantly higher in the diet-exposed group than in mice given BPA-*d*_6_ as a single bolus. In addition, concentrations of unconjugated or active BPA-*d*_6_ in the diet group were higher after 7 days of dietary exposure than at 24 hr after exposure. One untested explanation is that chronic exposure to BPA might eventually compromise metabolizing capacity of the liver, as suggested previously (Hanioka et al. 2008), leading to progressively elevated concentrations of active BPA. Variation in animal feeding habits might also account for differences between diet compared with bolus exposure, although there is no reason to presume that mice would exhibit different feeding patterns after 7 days versus 1 day of BPA-*d*_6_ exposure, particularly as the samples were collected at the same time of day (1900 hours). Another consideration is that the stage of the estrous cycle might influence BPA metabolism and accumulation. However, in a previous study Nepomnaschy et al. (2009) suggested that menstrual cycle stage did not influence urinary BPA concentrations in samples from 60 women taken 2 and 4 weeks apart. In short, the reason why circulating unconjugated BPA-*d*_6_ increased over time after diet exposure eludes us, but studies are currently under way with radioactive BPA to determine where the ingested BPA becomes concentrated.

## Conclusions

Our data highlight possible limitations of single oral bolus administration of BPA, the experimental design used for the majority of studies examining the pharmacokinetics of BPA exposure in both animal models and humans (Doerge et al. 2010a, 2010b; Taylor et al. 2011; Vandenberg et al. 2009). Results of the present study suggest that the presence of food may increase internal exposure to bioactive BPA, possibly by an inhibitory effect on first-pass (presystemic) elimination, and thus diet exposure is presumably the more relevant way of modeling the natural route of contact to BPA that occurs in humans. In contrast, experiments using single oral bolus exposure may not only underestimate exposure to bioactive BPA in serum but also lead to inaccurate conclusions concerning long-term concentrations of active BPA in serum or plasma of animals and humans. Our data may explain how although humans can rapidly eliminate BPA when it is provided as a single bolus (Volkel et al. 2002), continuous external BPA exposure appears to lead to sustained concentrations that are detectable in serum or plasma of humans who have not been knowingly exposed to this endocrine-disrupting chemical (FDA 2008; Vandenberg et al. 2007; vom Saal et al. 2007). We conclude that exposure through the diet provides a better approach for assessing the impact of BPA on internal organ systems than delivery as a single bolus.

## Supplemental Material

(356 KB) PDF

## References

[r1] Bailin PS, Byrne M, Lewis S, Liroff R (2008). Public Awareness Drives Market for Safer Alternatives: Bisphenol A Market Analysis Report.. http://www.iehn.org/publications.reports.bpa.php.

[r2] Balakrishnan B, Henare K, Thorstensen EB, Ponnampalam AP, Mitchell MD (2010). Transfer of bisphenol A across the human placenta.. Am J Obstet Gynecol.

[r3] Biedermann S, Tschudin P, Grob K. (2010). Transfer of bisphenol A from thermal printer paper to the skin.. Anal Bioanal Chem.

[r4] Calafat AM, Ye X, Wong LY, Reidy JA, Needham LL (2008). Exposure of the U.S. population to bisphenol A and 4-*tertiary*-octylphenol: 2003–2004.. Environ Health Perspect.

[r5] Cox KH, Gatewood JD, Howeth C, Rissman EF (2010). Gestational exposure to bisphenol A and cross-fostering affect behaviors in juvenile mice.. Horm Behav.

[r6] Dekant W, Volkel W. (2008). Human exposure to bisphenol A by biomonitoring: methods, results and assessment of environmental exposures.. Toxicol Appl Pharmacol.

[r7] Doerge DR, Twaddle NC, Vanlandingham M, Fisher JW (2010a). Pharmacokinetics of bisphenol A in neonatal and adult Sprague-Dawley rats.. Toxicol Appl Pharmacol.

[r8] Doerge DR, Twaddle NC, Woodling KA, Fisher JW (2010b). Pharmacokinetics of bisphenol A in neonatal and adult rhesus monkeys.. Toxicol Appl Pharmacol.

[r9] Dolinoy DC, Huang D, Jirtle RL (2007). Maternal nutrient supplementation counteracts bisphenol A-induced DNA hypomethylation in early development.. Proc Natl Acad Sci USA.

[r10] Environment Canada (2008). Screening Assessment for the Challenge Phenol, 4,4’-(1-methylethylidene) bis- (Bisphenol A). Chemical Abstracts Service Registry Number 80-05-7.. http://www.ec.gc.ca/substances/ese/eng/challenge/batch2/batch2_80-05-7.cfm.

[r11] FDA (Food and Drug Administration) (2008). Food and Drug Administration Draft Assessment of Bisphenol A for Use in Food Contact Applications, 14 August 2008.. http://www.fda.gov/ohrms/dockets/AC/08/briefing/2008-0038b1_01_02_FDA%20BPA%20Draft%20Assessment.pdf.

[r12] Food and Agriculture Organization of the United Nations and the World Health Organization (2010). Joint FAO/WHO Expert Meeting to Review Toxicological and Health Aspects of Bisphenol A.. http://www.who.int/foodsafety/chem/chemicals/bisphenol_release/en/index.html.

[r13] Galloway T, Cipelli R, Guralnik J, Ferrucci L, Bandinelli S, Corsi AM (2010). Daily bisphenol A excretion and associations with sex hormone concentrations: results from the InCHIANTI adult population study.. Environ Health Perspect.

[r14] Gies A, Heinzow B, Dieter HH, Heindel J (2009). Bisphenol A workshop of the German Federal Environment Agency—March 30–31, 2009: work group report: public health issues of bisphenol A.. Int J Hyg Environ Health.

[r15] Hanioka N, Takeda Y, Tanaka-Kagawa T, Hayashi K, Jinno H, Narimatsu S. (2008). Interaction of bisphenol A with human UDP-glucuronosyltransferase 1A6 enzyme.. Environ Toxicol.

[r16] He Y, Miao M, Herrinton LJ, Wu C, Yuan W, Zhou Z (2009). Bisphenol A levels in blood and urine in a Chinese population and the personal factors affecting the levels.. Environ Res.

[r17] Inoue H, Tsuruta A, Kudo S, Ishii T, Fukushima Y, Iwano H (2005). Bisphenol A glucuronidation and excretion in liver of pregnant and nonpregnant female rats.. Drug Metab Dispos.

[r18] Institute of Laboratory Animal Resources (1996). Guide for the Care and Use of Laboratory Animals. 7th ed.

[r19] Kavaliers M, Hirst M, Teskey GC (1985). Nocturnal feeding in the mouse—opiate and pineal influences.. Life Sci.

[r20] Kurebayashi H, Betsui H, Ohno Y. (2003). Disposition of a low dose of ^14^C-bisphenol A in male rats and its main biliary excretion as BPA glucuronide.. Toxicol Sci.

[r21] Matsumoto A, Kunugita N, Kitagawa K, Isse T, Oyama T, Foureman GL (2003). Bisphenol A levels in human urine.. Environ Health Perspect.

[r22] Nepomnaschy PA, Baird DD, Weinberg CR, Hoppin JA, Longnecker MP, Wilcox AJ (2009). Within-person variability in urinary bisphenol A concentrations: measurements from specimens after long-term frozen storage.. Environ Res.

[r23] Padmanabhan V, Siefert K, Ransom S, Johnson T, Pinkerton J, Anderson L (2008). Maternal bisphenol-A levels at delivery: a looming problem?. J Perinatol.

[r24] Pottenger LH, Domoradzki JY, Markham DA, Hansen SC, Cagen SZ, Waechter JM (2000). The relative bioavailability and metabolism of bisphenol A in rats is dependent upon the route of administration.. Toxicol Sci.

[r25] Sakamoto H, Yokota H, Kibe R, Sayama Y, Yuasa A. (2002). Excretion of bisphenol A-glucuronide into the small intestine and deconjugation in the cecum of the rat.. Biochim Biophys Acta.

[r26] Stahlhut RW, Welshons WV, Swan SH (2009). Bisphenol A data in NHANES suggest longer than expected half-life, substantial non-food exposure, or both.. Environ Health Perspect.

[r27] Tam YK (1993). Individual variation in first-pass metabolism.. Clin Pharmacokinet.

[r28] Taylor JA, Vom Saal FS, Welshons WV, Drury B, Rottinghaus G, Hunt PA (2011). Similarity of bisphenol A pharmacokinetics in rhesus monkeys and mice: relevance for human exposure.. Environ Health Perspect.

[r29] Teeguarden JG Calafat AM Ye X Doerge DR Churchwell MI Gunawan R 2011 24-Hour human urine and serum profiles of bisphenol A during high dietary exposure. Toxicol Sci doi: [Online 24 June 2011] 10.1093/toxsci/kfr160 21705716

[r30] Tominaga T, Negishi T, Hirooka H, Miyachi A, Inoue A, Hayasaka I (2006). Toxicokinetics of bisphenol A in rats, monkeys and chimpanzees by the LC-MS/mS method.. Toxicology.

[r31] Vandenberg LN, Chahoud I, Heindel JJ, Padmanabhan V, Paumgartten FJ, Schoenfelder G (2010a). Urinary, circulating, and tissue biomonitoring studies indicate widespread exposure to bisphenol A.. Environ Health Perspect.

[r32] Vandenberg LN, Chahoud I, Padmanabhan V, Paumgartten FJ, Schoenfelder G (2010b). Biomonitoring studies should be used by regulatory agencies to assess human exposure levels and safety of bisphenol A.. Environ Health Perspect.

[r33] Vandenberg LN, Hauser R, Marcus M, Olea N, Welshons WV (2007). Human exposure to bisphenol A (BPA).. Reprod Toxicol.

[r34] Vandenberg LN, Maffini MV, Sonnenschein C, Rubin BS, Soto AM (2009). Bisphenol-A and the great divide: a review of controversies in the field of endocrine disruption.. Endocr Rev.

[r35] Volkel W, Colnot T, Csanady GA, Filser JG, Dekant W (2002). Metabolism and kinetics of bisphenol A in humans at low doses following oral administration.. Chem Res Toxicol.

[r36] vom Saal FS, Akingbemi BT, Belcher SM, Birnbaum LS, Crain DA, Eriksen M (2007). Chapel Hill bisphenol A expert panel consensus statement: integration of mechanisms, effects in animals and potential to impact human health at current levels of exposure.. Reprod Toxicol.

[r37] Wilkinson GR (1997). The effects of diet, aging and disease-states on presystemic elimination and oral drug bioavailability in humans.. Adv Drug Deliv Rev.

[r38] Willhite CC, Ball GL, McLellan CJ (2008). Derivation of a bisphenol A oral reference dose (RfD) and drinking-water equivalent concentration.. J Toxicol Environ Health B Crit Rev.

