# Supplementary material for: Comparison of Serum Bisphenol A Concentrations in Mice Exposed to Bisphenol A through the Diet versus Oral Bolus Exposure
Source: Environ Health Perspect. 2011 Jun 6;119(9):1260–5. doi: 10.1289/ehp.1003385 (PMC3230401; doi:10.1289/ehp.1003385)
Supplement: (356 KB) PDF [file ehp.1003385.s001.pdf]

Supplemental Material

Comparison of Serum Bisphenol A Concentrations in Mice Exposed to Bisphenol A through the Diet versus Oral Bolus Exposure

Paizlee T. Sieli[1,2], Eldin Jašarević[2,3,4], Denise A. Warzak[1,2], Jiude Mao[5], Mark R. Ellersieck[6], Chunyang Liao[7], Kurunthachalam Kannan[7], Séverine H. Collet[8], Pierre-Louis Toutain[8], Frederick S. vom Saal[3], and Cheryl S. Rosenfeld[1,2]

[1]Biomedical Sciences, University of Missouri, Columbia, MO 65211 USA
[2]Bond Life Sciences Center, University of Missouri, Columbia, MO 65211 USA
[3]Biological Sciences, University of Missouri, Columbia, MO 65211 USA
[4]Interdisciplinary Neuroscience Program, University of Missouri, Columbia, MO 65211 USA
[5]Animal Sciences, University of Missouri, Columbia, MO 65211 USA
[6]Agricultural Experiment Station, University of Missouri, Columbia, MO 65211 USA
[7]Wadsworth Center, New York Department of Health, and Department of Environmental Health Sciences, School of Public Health, State University of New York at Albany, Albany, New York 12201-0509, USA.
[8] INRA; UMR 1331 TOXALIM (Research Centre in Food Toxicology); Université de Toulouse; INPT; ENVT; F-31076 Toulouse, France

Communicating Author**:** Cheryl S. Rosenfeld, D.V.M., Ph.D., Departments of Biomedical and Bond Life Sciences Center, University of Missouri, 440F Bond Life Sciences Center, 1201 E. Rollins Rd., Columbia, MO 65211. Email: rosenfeldc@missouri.edu, Phone: (573) 882-5132, and Fax: (573) 884-9395.

Running Title: Serum BPA Concentrations in Diet-Exposed Mice

Key Words**:** Bioavailability, BPA, Chronic Exposure, Deconvolution Analysis, Endocrine Disruptor, Food Effect, Oral Bolus, and Pharmokinetic Analysis

Table of Contents

Section	Section Heading	Page
1.0	BPA-d_6 Treatments	3
2.0	Analysis of Unconjugated and Conjugated BPA-d_6 in Mouse Serum Samples	3
3.0	Pharmokinetic Analysis	5
3.1	Non-compartmental analysis of total and unconjugated BPA-d_6 concentrations after oral and diet exposure	5
3.2	Compartmental analysis of unconjugated BPA-d_6 concentrations after oral bolus exposure	6
3.3	Deconvolution Analysis	7
4.0	References	8
**Tables**		
1	Components of AIN-93G control diet with 7% corn oil from Harlan-Teklad	9
2	Components of BPA-d_6 diet (100mg/kg feed weight) from Harlan-Teklad	10
3	Definition of the different pharmacokinetic parameters computed by using a non compartmental analysis	11
4	Pharmacokinetic parameters of unconjugated and total BPA-d_6 obtained by using a non compartmental data analysis in mice, BPA-d_6 dose of 20 mg/kg by oral route of exposure	12
5	Pharmacokinetic parameters of unconjugated and total BPA-d_6 obtained by a non compartmental data analysis in mice exposed to BPA-d_6 through the diet	14
6	Pharmacokinetic parameters of unconjugated BPA-d_6 obtained by compartmental data analysis in mice, BPA-d_6 dose of 20 mg/kg by oral bolus route of exposure	15
7	Deconvolution results giving input rate (mg/h) for step of 0.084 h (5.04 min), corresponding cumulative input in mg and in percentage of the total input rate	16
**Figures**		
1	Semi-logarithmic plot of observed (○) and fitted (-) unconjugated BPA-d_6 concentrations *versus* time (h) after oral bolus at 20 mg/kg BW	19
2	Time *versus* weighted residual pooled serum concentrations of BPA-d_6 for a monocompartmental model without lag-time	20
3	Curve representing the mean unconjugated BPA-d_6 serum concentration profile between 0 and 11 h after the beginning of the meal obtained by a smoothing procedure	21
4	Input rate (mg/h) versus time (h) of BPA-d_6 in mice fed with the supplemented BPA-d_6 diet	22

1.0 BPA-d_6 Treatments

Body weight was measured and food consumption from the beginning of the dosing period was calculated at each timepoints. Blood samples were collected by cardiac puncture in polypropylene (PP) syringes, placed in PP microcentrifuge tubes, and centrifuged twice (15 min at 13,800 g) (Mikro 22R Microcentrifuge, Hettich Zentrifugen, Germany) for serum collection. The serum samples were stored at -20°C. Individual samples within the same timepoint and treatment group that provided less than the minimum 400 μL needed for the BPA-d_6 analysis were pooled to yield 9 negative control samples. The number of pooled samples for mice given the oral bolus of BPA-d_6 sacrificed at each time was: 1 h, n= 3; 4 h, n= 4; 6 h, n= 4; 11 h, n=4; 24 h, n=6; 7 d, n=4. For the BPA-d_6 diet-exposed group, the number of pooled samples for each timepoint was: 1 h, n= 6; 4 h, n= 4; 6 h, n= 4; 11 h, n=4; 24 h, n=6; 7 d, n=5. The samples were shipped on dry ice to the Wadsworth Center, Albany, NY for analysis.

2.0 Analysis of Unconjugated and Conjugated BPA-d_6 in Mouse Serum Samples

To assess "freely available" BPA, i.e. that fraction that had not been metabolized to its water-soluble, glucuronyl derivative, each sample was allowed to thaw at room temperature for 30 min and transferred into a 15 mL PP tube. An internal standard (5 ng of deuterated 16-bisphenol A (BPA-d_{16}) was added. Five mL of ethyl acetate was then added, and the mixture shaken in an orbital shaker for 30 min. After centrifugation at 4500 g for 3 min (Eppendorf Centrifuge 5804, Hamburg, Germany), the ethyl acetate layer was transferred to a clean PP tube, the residue extracted two more times with 3.5 mL of ethyl acetate by shaking, and the extracts were combined. The extracts were mixed with 3.5 mL of milli-Q water and centrifuged at 5000 rpm for 3 min (Eppendorf Centrifuge 5804). The ethyl acetate layer was collected, dried under a

gentle stream of nitrogen and reconstituted in 0.5 mL of methanol. This fraction that was soluble in ethyl acetate was considered freely available BPA-d_6.

After addition of the internal control, the second aliquot of thawed serum was digested with 1 mL of 2 μL/mL *β*-glucuronidase that also has aryl-sulfatase activity (from *Helix pomatia*, 145700 unit/mL, Sigma, St Louis, MO) at 37ºC for 12 h. The digested sample was processed as described above and referred to as total BPA-d_6, which includes the free, i.e. ethyl acetate soluble, plus the conjugated BPA-d_6.

The BPA-d_6 serum concentrations in samples were measured by a procedure similar to that described earlier, but with some modifications (Padmanabhan et al. 2008). Analyte separation and detection were carried out by using an Agilent 1100 series HPLC interfaced with an Applied Biosystems API 2000 electrospray MS/MS mass spectrometer (Applied Biosystems, Foster City, CA). Samples (10 μL) of the extract were injected onto an analytical column (Betasil® C18, 100 x 2.1 mm column; Thermo Electron Corporation, Waltham, MA), connected in series to a Javelin® guard column (Betasil® C18, 20 x 2.1 mm). The mobile phase was comprised of a gradient of methanol/water (initial 25% methanol; final 99% methanol; 4 min). Flow was maintained under the latter conditions for 10 min before reversing to the initial starting condition. The flow rate and the column temperature were 300 μL/min and 25°C respectively. The MS/MS was operated in the electrospray negative ion mode. Instrument parameters were optimized to transmit the [M-H] ion before fragmentation to one or more product ions. Cone voltage and collision energies were 30 V and 25 V, respectively. Capillary voltage was 4.5 KV,

and desolvation temperature 400° C. Data were acquired by using multiple reaction monitoring (MRM) for the transitions of 233>215 for BPA-d_6 and 241>223 for BPA-d_{16}.

Quality assurance and control parameters included validation of the method by spiking BPA-d_{16} into the sample matrices and passing through the entire analytical procedure to calculate recoveries of BPA-d_{16} through the analytical method. The matrix spike recovery was 109 % (106-111%) with a standard deviation of 3.5%. A procedural blank, containing milli-Q water in place of serum was analyzed in parallel with the samples to check for interferences that would correlate with the target compound or laboratory contamination. Trace concentrations of BPA present in such blanks (<0.01 ng) were subtracted from sample values for determining the concentrations in samples. The limit of detection, i.e. sensitivity of the assay, was 0.1 ng/mL, which was calculated as twice that of the valid "lowest acceptable calibration standard". The reported concentrations of BPA-d_6 for each sample were corrected, based on the recovery value of the surrogate standard, BPA-d_{16} (i.e., isotope dilution). The BPA-d_6 standard spiked into sample matrices and passed through the entire analytical procedure yielded a mean recovery of 117%. An external calibration curve prepared by injecting 10 µL of 0.05, 0.1, 0.2, 0.5, 1, 2, 5, 10, 20, and 50 ng/mL standards of BPA-d_6 and BPA-d_{16} yielded a calibration coefficient of 0.99.

3.0 Pharmacokinetic analysis

3.1 Non-compartmental analysis of total and unconjugated BPA-d_6 concentrations after oral and diet exposure

Extrapolation to infinity to obtain $AUC_{(0\text{-infinity})}$ was calculated by dividing the last observed quantifiable serum concentrations by the slope of the terminal phase as estimated by linear regression by using the best fit option of WinNonlin.

Mean Residence Time (MRT), which refers to the average total time BPA-d_6 molecules of a given BPA-d_6 dose spend in the body, was obtained with and without extrapolation to infinity by using statistical moments (Gibaldi and Perrier, 1982). MRT can be viewed as the arithmetic mean of times that each BPA-d_6 molecule spends in the body, and it is a metric of persistency of BPA in the body because it is a stochastic view of BPA-d_6 pharmacokinetics in the body.

The apparent oral clearance (Cl/F) was obtained by dividing the administered BPA-d_6 dose by the corresponding $AUC_{(0\text{-}Clast)}$, C_{last} being the 24h serum BPA-d_6 concentration. The sparse data option of WinNonlin was used, allowing computation of the different standard errors (SE) associated with estimated parameters. Definitions of the different computed parameters are given in Supplemental Material, Table 3.

Results of the NCA for unconjugated and total after oral bolus and diet exposure are presented in Supplemental Material, Tables 4 and 5.

3.2 Compartmental analysis of unconjugated BPA-*d*₆ concentrations after oral bolus exposure

The terminal half-life ($T_{1/2}$) was defined as $\frac{\ln(2)}{K_{10}}$ and T_{max}, time at the maximal concentration (C_{max}) as $\frac{\ln(K_{01}/K_{10})}{K_{01}-K_{10}}$. The area under the concentration curve *versus* time (AUC) from time zero to the last detectable concentration was calculated as $AUC = \frac{D}{\frac{V}{F}\times K_{10}}$ with $\frac{V}{F}\times K_{10}$ being

the apparent clearance (Clearance/F). The estimated final BPA parameters are presented in Supplemental Material, Table 6.

3.3 Deconvolution analysis

The curve representing mean unconjugated BPA-d_6 serum concentrations over time (0-11 h) was presented with automatic smoothing procedure in Supplemental Material, Figure 3, and these are the data that were analyzed by deconvolution.

Deconvolution computed for each discretization step (here 0.08h or 5.04 min, Supplemental Material, Table 7) was the amount of BPA-d_6 that entered the blood from the feed. For example, at 1.93 h, the BPA-d_6 input rate was 1.23 mg/h and at that time the total amount of BPA-d_6 that had entered the bloodstream was 0.448 mg *in toto* representing 2.87 % of the dose that was actually bioavailable at the end period of observation. The shape of the input rate during the night is given by Supplemental Material, Figure 4. Visual inspection of the curve indicates that after time 0 (19:00 h in the trial), there was a lag time of about 1 h before BPA-d_6 entered the bloodstream. This delay was presumably due to food consumption and gastrointestinal transit times, release of BPA-d_6 from food, and BPA-d_6 absorption. Thereafter, the BPA-d_6 input rate increased rapidly to reach a maximum value at 4.2 h (23:12 h in the trial) and then returned to base line after a delay of 7 to 8 h, after the beginning of the evening meal (i.e at about 02:00 to 03:00 h the next day). The cumulative input rate indicates that 50 % of the bioavailable BPA-d_6 dose had entered the bloodstream at 4.11h (23:00 h) and that 90 % of the dose gained access to blood at 5.46 h after the beginning of the meal, i.e. before 1:00 AM the next day. The total

estimated absorbed dose between 0 and 11 h by deconvolution analysis was 15.6 mg/kg over the first 11 h.

4.0 References

Gibaldi, M. and Perrier, D. (1982) *Pharmacokinetics*, 2nd edn., Marcel Dekker, New York.

Padmanabhan V, Siefert K, Ransom S, Johnson T, Pinkerton J, Anderson L, et al. 2008. Maternal bisphenol-A levels at delivery: a looming problem? J Perinatol 28(4): 258-263.

Supplemental Material, Table 1: Components of AIN-93G control diet with 7% corn oil from Harlan-Teklad

**Diet Component**		**g/kg**
Casein		200.0
L-Cystine		3.0
Corn Starch		397.386
Maltodextrin		132.0
Sucrose		100.0
Corn Oil		70.0
Cellulose		35.0
Mineral Mix, AIN-93G-MX (94046)		35.0
Vitamin Mix, AIN-93-VX (94047)		10.0
Choline Bitartrate		2.5
TBHQ, Antioxidant		0.014
**Selected Nutrient Information**		
	**% by Weight**	**% kcal**
**Protein**	17.7	18.8
**Carbohydrate**	60.1	63.9
**Fat**	7.2	17.2

Kcal/g **3.8**

Supplemental Material, Table 2: Components of BPA-d_6 diet (100mg/kg feed weight) from Harlan-Teklad

**Diet Component**		**g/kg**
Casein		200.0
L-Cystine		3.0
Corn Starch		397.386
Maltodextrin		132.0
Sucrose		90.0
Corn Oil		70.0
Cellulose		35.0
Mineral Mix, AIN-93G-MX (94046)		35.0
Vitamin Mix, AIN-93-VX (94047)		10.0
Choline Bitartrate		2.5
TBHQ, Antioxidant		0.014
BPA-d_6 Mixture (1% in Sucrose)		10.0
Pink Food Color		0.1
**Selected Nutrient Information**		
	**% by Weight**	**% kcal**
**Protein**	17.7	18.8
**Carbohydrate**	60.0	63.9
**Fat**	7.2	17.3

Kcal/g 3.8

Supplemental Material, Table 3: Definition of the different pharmacokinetic parameters computed by using a non-compartmental analysis

**Pharmacokinetic Parameter**	**Definition**
AUC_%Extrap_obs	Percentage of AUCINF_obs that is due to extrapolation from Tlast to infinity; extrapolation done with lambda_z; AUC: Area under the curve
AUCINF_obs	AUC from time of dosing (0) to infinity; extrapolation with the last quantifiable (i.e. above LOQ) concentration divided by the terminal slope (lambda_z)
AUClast	AUC from time of dosing (0) to the time of the last quantifiable concentration
Cl_F_obs	Apparent total serum clearance for extravascular administration (or oral clearance) calculated from AUCINF_obs
Cl_F_last	Apparent total serum clearance for extravascular administration (or oral clearance) calculated from AUClast
Clast	Concentration observed at Tlast
Cmax	Maximal serum BPA concentration
HL_Lambda_z	Terminal half-life (ln(2)/terminal slope); best fit option of WinNonlin
MRTINF_obs	Mean Residence Time (MRT) extrapolated to infinity using the last quantifiable serum concentration for extrapolation
MRTlast	Mean Residence Time (MRT) from time of dosing to the last quantifiable serum BPA concentration
Tlast	Time of last quantifiable serum concentration
Tmax	Time of maximal serum BPA concentration

**Additional output**	**Definition**
Corr_XY	Correlation between time (X) and log concentration (Y) for the points used in estimation of the terminal slope (lambda_z)
SE_AUClast	Standard error (SE) associated with AUClast estimate for sparse data analysis in WinNonlin (mouse data)
SE_Cmax	Standard error (SE) associated with Cmax estimate for sparse data analysis in WinNonlin (mouse data)

Supplemental Material, Table 4: Pharmacokinetic parameters of unconjugated and total BPA-d_6 obtained by a non compartmental data analysis in mice, BPA-d_6 dose of 20 mg/kg by oral route of exposure

**Parameter**	**Units**	**Estimate for Unconjugated** BPA-d_6	**Estimate for Total** BPA-d_6
Rsq		0.9946	0.9683
Rsq_adjusted		0.9893	0.9366
Corr_XY		-0.9973	-0.9840
No_points_lambda_		3.0000	3.0000
Lambda_z	1/hr	0.1356	0.1758
Lambda_z_lower	hr	6.0000	6.0000
Lambda_z_upper	hr	24.0000	24.0000
HL_Lambda_z	hr	5.1109	3.9435
Tlag	hr	0.0000	0.0000
Tmax	hr	1.0000	6.0000
Cmax	ng/mL	21.0000	1636.5000
SE_Cmax	ng/mL	3.9038	642.6338
Tlast	hr	24.0000	24.0000
Clast	ng/mL	1.2533	80.1000
AUClast	hr*ng/mL	200.9942	21979.3583
SE_AUClast	hr*ng/mL	20.6535	3813.4673
AUCall	hr*ng/mL	200.9942	21979.3583
SE_AUCall	hr*ng/mL	20.6535	3813.4673
AUCINF_obs	hr*ng/mL	210.2355	22435.0636
AUCINF_D_obs	hr*kg*ng/mL/n	0.0000	0.0006
AUC_%Extrap_obs	%	4.3957	2.0312
Vz_F_obs	mL/kg	701443.2838	8984.5299
Cl_F_obs	mL/hr/kg	95131.4064	1579.2244
AUCINF_pred	hr*ng/mL	210.5049	22478.8676
AUCINF_D_pred	hr*kg*ng/mL/n	0.0000	0.0006
AUC_%Extrap_pred	%	4.5181	2.2221
Vz_F_pred	mL/kg	700545.6892	8967.0220
Cl_F_pred	mL/hr/kg	95009.6725	1576.1470
AUMClast	hr*hr*ng/mL	1462.5800	175837.4333
AUMCINF_obs	hr*hr*ng/mL	1752.5126	189366.9594

AUMC_%Extrap_ob	%	16.5438	7.1446
AUMCINF_pred	hr*hr*ng/mL	1760.9636	190667.4665
AUMC_%Extrap_pr	%	16.9443	7.7780
MRTlast	hr	7.2767	8.0001
MRTINF_obs	hr	8.3359	8.4407
MRTINF_pred	hr	8.3654	8.4821

Supplemental Material, Table 5: Pharmacokinetic parameters of unconjugated and total BPA-d_6 obtained by a non compartmental data analysis in mice exposed to BPA-d_6 through the diet

		**Ingested Dose (13mg/kg BW)**		**Corrected Dose (20mg/kg BW)**	
**Parameter**	**Units**	**Estimate for Unconjugated BPA-d_6**	**Estimate for Total BPA-d_6**	**Estimate for Unconjugated BPA-d_6**	**Estimate for Total BPA-d_6**
Tmax	h	6.0000	6.0000	6.0000	6.0000
Cmax	ng/mL	18.8150	802.2500	28.9460	1234.2300
SE_Cmax	ng/mL	4.3763	126.5717	6.7327	194.7239
Cmax_D	kg*ng/mL/ng	0.0000	0.0000	0.0000	0.0001
Tlast	h	24.0000	24.0000	24.0000	24.0000
Clast	ng/mL	6.9417	193.9333	10.6782	296.8600
AUClast	h*ng/mL	147.8358	11547.3200	227.4235	17755.3500
SE_AUClast	h*ng/mL	26.7339	1219.5527	41.1317	1874.6794
AUCall	h*ng/mL	147.8358	11547.3200	227.4235	17755.3500
SE_AUCall	h*ng/mL	26.7339	1219.5527	41.1317	1874.6794
AUCINF_obs	h*ng/mL	359.5439	13867.3357	553.0144	21293.9150
AUCINF_D_obs	h*kg*ng/mL/ng	0.0000	0.0004	0.0000	0.0016
AUC_%Extrap_obs	%	58.8824	16.7301	58.8757	16.6177
Vz_F_obs	mL/kg	1102719.5094	30564.4501	716774.4759	7277.1835
Cl_F_obs	mL/h/kg	36156.9223	2554.9248	23507.5252	610.5030
AUCINF_pred	h*ng/mL	312.0034	13993.5203	479.9031	21487.2824
AUCINF_D_pred	h*kg*ng/mL/ng	0.0000	0.0004	0.0000	0.0017
AUC_%Extrap_pred	%	52.6172	17.4810	52.6105	17.3681
Vz_F_pred	mL/kg	1270742.7590	30288.8394	825972.2273	7211.6950
Cl_F_pred	mL/h/kg	41666.2141	2531.8861	27088.8036	605.0090
AUMClast	h*h*ng/mL	1830.9650	123052.8533	2816.6625	189078.0700
AUMCINF_obs	h*h*ng/mL	13368.6633	206487.4734	20558.5290	316183.2496
AUMC_%Extrap_obs	%	86.3041	40.4066	86.2993	40.1998
AUMCINF_pred	h*h*ng/mL	10777.7943	211025.4451	16574.5991	323129.0041
AUMC_%Extrap_pred	%	83.0117	41.6881	83.0062	41.4853
MRTlast	h	12.3851	10.6564	12.3851	10.6491
MRTINF_obs	h	37.1823	14.8902	37.1754	14.8485
MRTINF_pred	h	34.5438	15.0802	34.5374	15.0382

Supplemental Material, Table 6: Pharmacokinetic parameters of unconjugated BPA-d_6 obtained by compartmental data analysis in mice, BPA-d_6 dose of 20 mg/kg by oral bolus route of exposure

Parameter	Units	Estimate	Std Error	CV%
V_F	mL/kg	873624.901968	115632.597853	13.24
Initial rate	1/hr	3.299308	5.038292	152.71
Terminal rate	1/hr	0.108765	0.018661	17.16
AUC	hr*ng/mL	210.483104	22.448839	10.67
Initial $_{t1/2}$	hr	0.210089	0.320501	152.56
Terminal $t_{1/2}$	hr	6.372909	1.092303	17.14
Tmax	hr	1.069499	1.183828	110.69
Cmax	ng/mL	20.379160	2.671239	13.11

Supplemental Material, Table 7: Deconvolution results giving input rate (mg/h) for step of 0.084 h (5.04 min), corresponding cumulative input (mg) and in percentage of the total input rate

Time (h)	Input Rate (mg/h)	Cumulative Input (mg)	Fraction Input
0.00	0.000000	0.000000	0.000000
0.08	0.000000	0.000000	0.000000
0.17	0.000000	0.000000	0.000000
0.25	0.000000	0.000000	0.000000
0.34	0.000000	0.000000	0.000000
0.42	0.000000	0.000000	0.000000
0.50	0.000000	0.000000	0.000000
0.59	0.000000	0.000000	0.000000
0.67	0.000000	0.000000	0.000000
0.76	0.000000	0.000000	0.000000
0.84	0.000000	0.000000	0.000000
0.92	0.000000	0.000000	0.000000
1.01	0.000164	0.000000	0.000000
1.09	0.021896	0.000682	0.000044
1.18	0.074875	0.004551	0.000292
1.26	0.152720	0.013951	0.000894
1.34	0.250350	0.030751	0.001970
1.43	0.363730	0.056435	0.003616
1.51	0.489640	0.092185	0.005907
1.60	0.625520	0.138940	0.008903
1.68	0.769330	0.197460	0.012652
1.76	0.919460	0.268320	0.017192
1.85	1.074600	0.352010	0.022555
1.93	1.233800	0.448900	0.028763
2.02	1.396100	0.559300	0.035836
2.10	1.561000	0.683430	0.043790
2.18	1.727800	0.821500	0.052637
2.27	1.896300	0.973650	0.062386
2.35	2.066100	1.140000	0.073045
2.44	2.236900	1.320700	0.084620
2.52	2.408500	1.515700	0.097116
2.60	2.580700	1.725100	0.110540
2.69	2.753400	1.949100	0.124890
2.77	2.926600	2.187600	0.140170
2.86	3.100000	2.440600	0.156380
2.94	3.273800	2.708200	0.173530
3.02	3.447700	2.990400	0.191610
3.11	3.621800	3.287200	0.210620
3.19	3.796000	3.598600	0.230580
3.27	3.970400	3.924700	0.251470
3.36	4.144800	4.265400	0.273300

3.44	4.319300	4.620800	0.296070
3.53	4.493800	4.990800	0.319780
3.61	4.668400	5.375500	0.344430
3.69	4.843000	5.774800	0.370020
3.78	5.017600	6.188800	0.396540
3.86	5.192200	6.617400	0.424010
3.95	5.366900	7.060800	0.452410
4.03	5.535200	7.518700	0.481750
4.11	5.632400	7.988100	0.511830
4.20	5.656300	8.462500	0.542230
4.28	5.621800	8.936400	0.572590
4.37	5.540800	9.405400	0.602640
4.45	5.422800	9.865900	0.632150
4.53	5.275300	10.315000	0.660940
4.62	5.104500	10.751000	0.688870
4.70	4.915000	11.172000	0.715830
4.79	4.710700	11.576000	0.741740
4.87	4.494600	11.963000	0.766500
4.95	4.269100	12.331000	0.790080
5.04	4.036100	12.679000	0.812430
5.12	3.797200	13.008000	0.833500
5.21	3.553500	13.317000	0.853280
5.29	3.306100	13.605000	0.871730
5.37	3.055700	13.872000	0.888850
5.46	2.802900	14.118000	0.904610
5.54	2.548200	14.343000	0.919010
5.63	2.292000	14.546000	0.932030
5.71	2.034500	14.728000	0.943670
5.79	1.776200	14.888000	0.953920
5.88	1.517000	15.026000	0.962780
5.96	1.257300	15.142000	0.970240
6.05	1.005600	15.237000	0.976320
6.13	0.800240	15.313000	0.981150
6.21	0.636810	15.373000	0.985000
6.30	0.506760	15.421000	0.988060
6.38	0.403270	15.459000	0.990500
6.47	0.320910	15.489000	0.992440
6.55	0.255370	15.513000	0.993990
6.63	0.203220	15.532000	0.995210
6.72	0.161720	15.547000	0.996190
6.80	0.128690	15.560000	0.996970
6.89	0.102410	15.569000	0.997590
6.97	0.081493	15.577000	0.998080
7.05	0.064850	15.583000	0.998470
7.14	0.051606	15.588000	0.998780
7.22	0.041067	15.592000	0.999030

7.31	0.032680	15.595000	0.999230
7.39	0.026006	15.597000	0.999390
7.47	0.020695	15.599000	0.999510
7.56	0.016468	15.601000	0.999610
7.64	0.013105	15.602000	0.999690
7.73	0.010429	15.603000	0.999750
7.81	0.008299	15.604000	0.999800
7.89	0.006604	15.604000	0.999840
7.98	0.005255	15.605000	0.999880
8.06	0.004182	15.605000	0.999900
8.15	0.003328	15.606000	0.999920
8.23	0.002648	15.606000	0.999940
8.31	0.002108	15.606000	0.999950
8.40	0.001677	15.606000	0.999960
8.48	0.001335	15.606000	0.999970
8.56	0.001062	15.606000	0.999980
8.65	0.000845	15.607000	0.999980
8.73	0.000673	15.607000	0.999980
8.82	0.000535	15.607000	0.999990
8.90	0.000426	15.607000	0.999990
8.98	0.000339	15.607000	0.999990
9.07	0.000270	15.607000	0.999990
9.15	0.000215	15.607000	0.999990
9.24	0.000171	15.607000	1.000000
9.32	0.000136	15.607000	1.000000
9.40	0.000108	15.607000	1.000000
9.49	0.000086	15.607000	1.000000
9.57	0.000068	15.607000	1.000000
9.66	0.000055	15.607000	1.000000
9.74	0.000043	15.607000	1.000000
9.82	0.000035	15.607000	1.000000
9.91	0.000027	15.607000	1.000000
9.99	0.000022	15.607000	1.000000
10.08	0.000017	15.607000	1.000000
10.16	0.000014	15.607000	1.000000
10.24	0.000011	15.607000	1.000000
10.33	0.000009	15.607000	1.000000
10.41	0.000007	15.607000	1.000000
10.50	0.000006	15.607000	1.000000
10.58	0.000004	15.607000	1.000000
10.66	0.000004	15.607000	1.000000
10.75	0.000003	15.607000	1.000000
10.83	0.000002	15.607000	1.000000
10.92	0.000002	15.607000	1.000000
11.00	0.000001	15.607000	1.000000

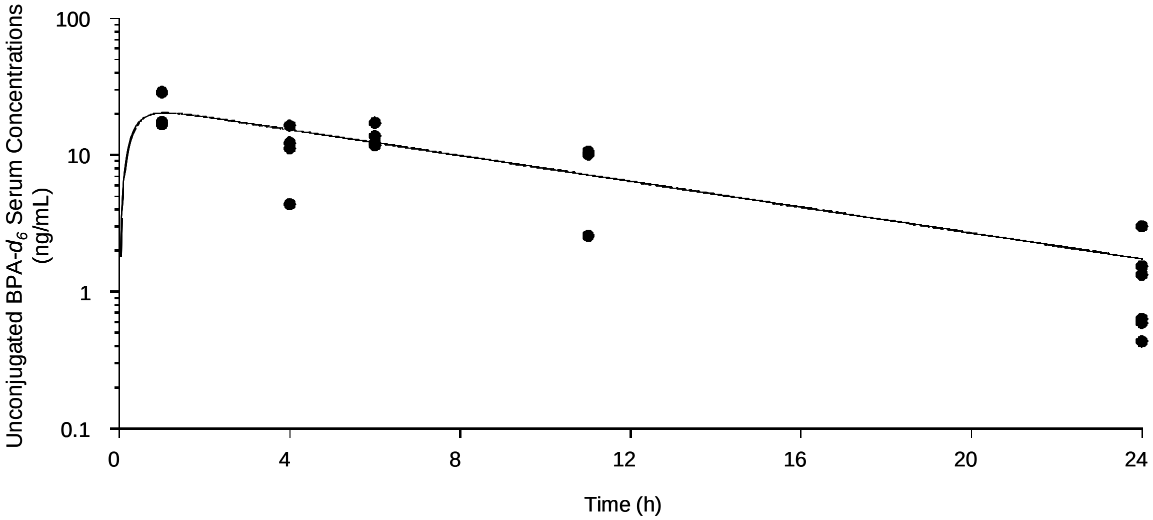

Supplemental Material, Figure 1: Semi-logarithmic plot of observed (○) and fitted (-) unconjugated BPA-d_6 concentrations *versus* time (h) after oral bolus at 20 mg/kg BW. Pooled data were fitted by using the so-called Bateman equation and weighted by the inverse of the observed value (1/Yobs).

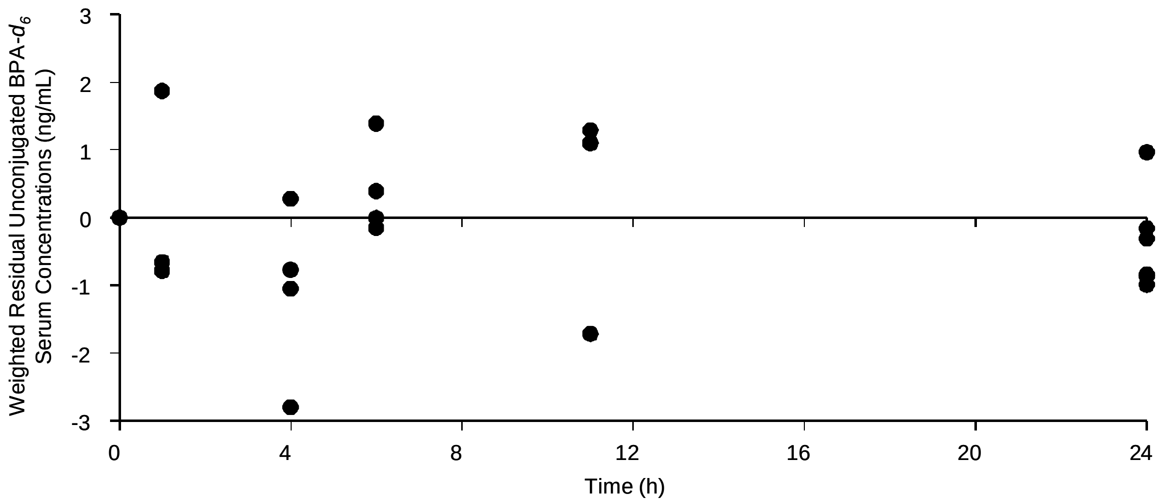

Supplemental Material, Figure 2: Time *versus* weighted residual pooled serum concentrations of BPA-d_6 for a monocompartmental model without lag-time. Visual inspection of the graph indicates appropriate scatter of residuals (no bias, homoscedasticity), and thus, the goodness of the fit of the model.

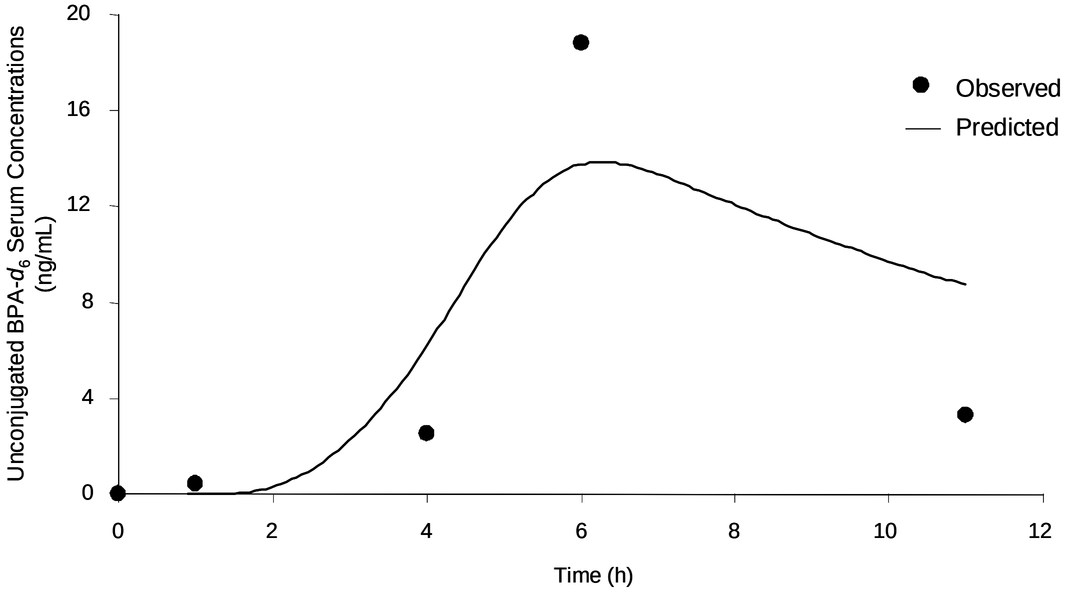

Supplemental Material, Figure 3: Curve representing the mean unconjugated BPA-d_6 serum concentration profile (ng/mL) between 0 and 11 h after the beginning of the meal obtained by a smoothing procedure (not a modelling).

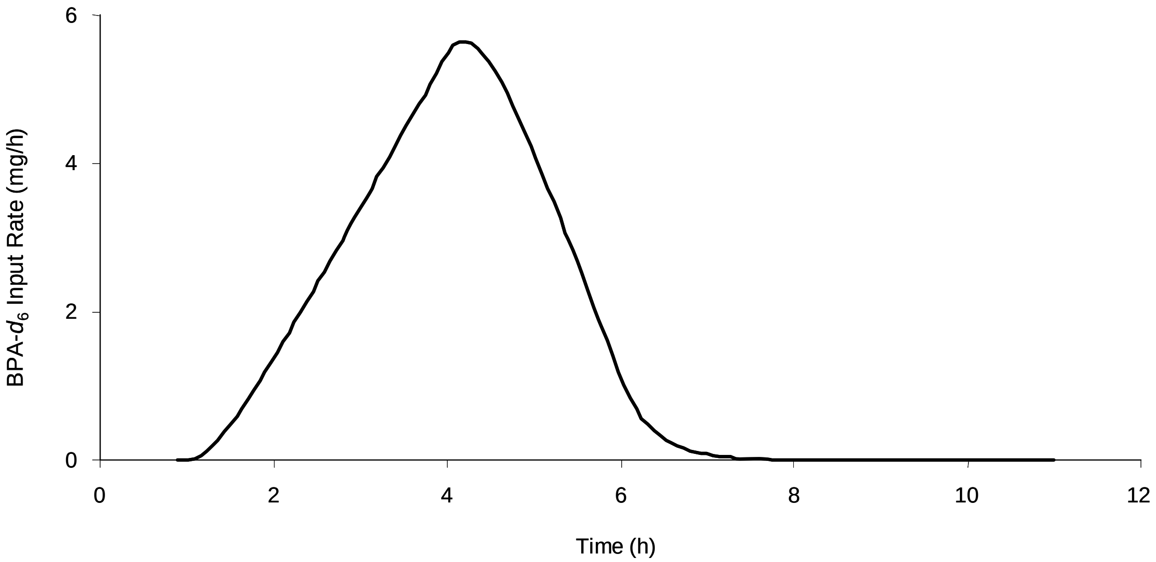

Supplemental Material, Figure 4: Input rate (mg/h) *versus* time (h) of BPA-d_6 in mice fed with the supplemented BPA-d_6 diet.
